# Production and Characterization of Large-Scale Recycled Newspaper Enhanced HDPE Composite Laminates

**DOI:** 10.3390/polym12040851

**Published:** 2020-04-07

**Authors:** Binwei Zheng, Litao Guan, Weiwei Zhang, Jin Gu, Dengyun Tu, Chuanshuang Hu

**Affiliations:** College of Materials and Energy, South China Agricultural University, Guangzhou 510642, China; hnzbw@stu.scau.edu.cn (B.Z.); zhangww@scau.edu.cn (W.Z.); gujin@scau.edu.cn (J.G.); tudengyun@scau.edu.cn (D.T.)

**Keywords:** recycled newspaper, full-scale, laminated composite, physical and mechanical properties

## Abstract

Recycled newspaper (NP)/high density polyethylene (HDPE) laminated composite can reach the physical and mechanical criteria for most industrial applications, which shows the potential of using solid-state waste paper in engineering materials. Herein, the effects of splicing pattern and size on the physical and mechanical properties of the laminated composite were investigated with the ultimate purpose to fabricate a large-scaleale composite. The laminated composite with a stair-like splicing had better physical and mechanical properties than that with a vertical splicing. An efficient stress transfer could be guaranteed when the distance between the two adjacent junctions were greater than a critical proportion of 1/32 of the length at longitudinal direction. The tensile and flexural properties of the large-scaleale composite with a stair-like splicing, which was fabricated at the splicing ratio of 1/32, were 109 ± 4.2 MPa (MOR), 9836 ± 411 MPa (MOE), 119 ± 7.1 MPa (MOR) and 10002 MPa ± 347 (MOE).

## 1. Introduction

As a degradable and environmentally friendly biomass material, recycled paper has been used to create bio-based products in recent decades, such as biogas [1], bioethanol [2], and biofertilizer [3]. Besides, high-value cellulosic products, carboxymethyl cellulose (CMC) [4], cellulose nanofibers (CNFs) [5], and cellulose nanocrystals (CNCs) [6] have also been developed successfully from recycled paper fiber because of their desired mechanical performance and functional application.

Recently, unshrouded recycled paper sheets were used to fabricate laminated composites based on their advantages of high consistency in quality, source, and mechanical properties. The influence of paper type, matrix selection, and fiber loading on the physical and mechanical properties of the composites have been studied [7,8,9,10]. High utilization ratios of waste paper and desired mechanical performances were achieved. It has been proven that it is an efficient way to utilize the recycled paper sheet as a laminar for enhanced laminated composites. However, so far the paper sheet based laminated composites manufactured in the laboratory were still small in size, which would limit its application in the aerospace and other engineering fields, where a large-scaleale is required [11,12,13,14]. In practice, for a large scale paper sheet laminated composite structure, all the paper sheet layers need laminar splices with the junction of different paper sheets in order to extend the dimensions of length and width. When a splicing structure occurs, it inevitably introduces defects at the splicing areas, which may pose serious stress concentration.

In the past decade, some kinds of splicing fiber reinforced laminated composites have been studied. Zhang et al. [15] investigated the failure modes of glass fiber reinforced 2D woven fabric with stair-like splicing laminate composites and found that the failure modes were from rapid damage to accumulating failure when the interval length increased from 4 to 12 mm and all fractures occur at the site of the splicing line due to the stress concentration. Chen et al. [16] studied the tensile failure characteristics and the stress distributions of unidirectional carbon fiber-reinforced laminate polymers (CFRP) with different splicing structures. The tensile strength decreased obviously after inducing the splicing structure into laminated composites. The initial fracture happened in the splicing areas and result in an interlaminar shear failure due to the stress concentration. Zhu et al. [17] analyzed the effects of ply splices at different positions on the tensile properties of unidirectional CFRP and the coupling between position of ply splicing by establishing a finite element model. Results showed that the ply splicing positions should be kept as far away from each other as possible to avoid coupling effects. However, there are no relative studies directly related to spliced recycled paper laminated composites. Besides, the stress concentration and coupling effects on the mechanical properties of splicing laminated composites should be considered when a splicing structure is introduced to the laminated composites. 

The objective of this work is to fabricate a large scale recycled newspaper (NP) / high density polyethylene (HDPE) laminated composites by incorporation of splicing method. The effects of the splicing shape, splicing length, and stacking orientations on the physical and mechanical properties of the composite were investigated. 

## 2. Materials and Methods

### 2.1. Materials

Non-oriented HDPE (density 0.95 g/cm^3^; MFR 0.24g/10min at 190 °C/2.16 kg; T_m_ 119 °C) films without any additives, which were extruded as mono-film, with a thickness of 12.8 μm were used as matrix, provided by local company (Charoen Pokphand Group, Guangzhou, China). The unshrouded NP sheets consisting of 20.9% CaCO_3_ and 79.1% cellulosic materials with the dimensions of 540 mm (L) × 780 mm (W) × 50 um (T) and a mass per unit area (grammage) of 45 g/m^2^, were recycled from daily life and used as reinforcement, which were provided by the Shanghai Securities News (Shanghai, China). Poly tetra fluoroethylene (PTFE) films were used as the demolding layer to facilitate the demolding process. 

### 2.2. Composite Fabrication

**Figure 1:**
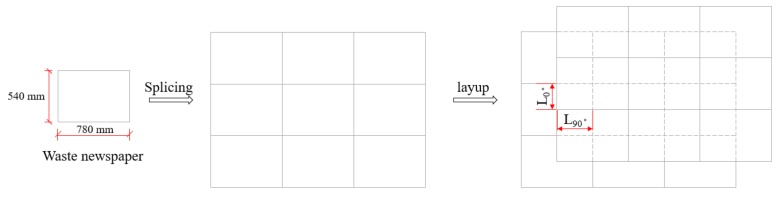
The diagram of laminates splicing and layup.

**Figure 2:**
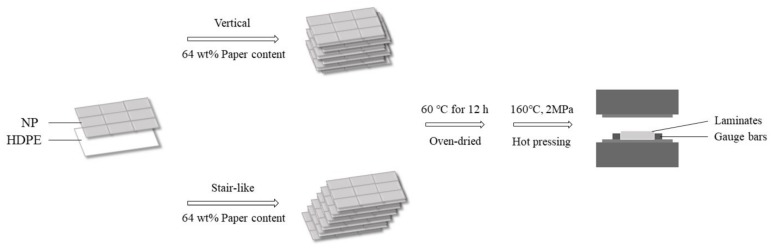
The fabrication procedure of laminated composite with the splicing junction.

**Figure 3:**
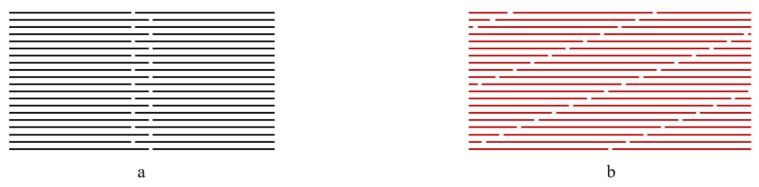
The diagram of (**a**) vertical and (**b**) stair-like splicing structures of the laminated composites.

NP sheets were stacked to form the splicing structure, which is depicted in Figure 1. The procedure how to fabricate the laminated composites with the splicing junction is shown in Figure 2. NP sheet is a typical orthotropic material to have different mechanical properties at length (90°) and width (0°) directions. The distances between the adjacent two junctions at the length and width direction were set to be one certain proportion of 1/128, 1/64, 1/32, 1/16, or 1/8 of NP length (90°) and width (0°). The splicing proportion and its certain size were shown in Table 1. The junction vertically repeated or had a stair pattern within the thickness of the laminated composites, which was depicted in Figure 3. 

Laminates were fabricated by stacking NP sheets and HDPE films alternately with the same direction, where paper content was 64 wt %. Then, laminates were hot-pressed for 25 min to ensure a sufficient compaction and infiltration. The heating temperature and pressure were set to 160 °C and 2 MPa, respectively. Besides, to investigate the effect of thickness on the physical and mechanical properties of the composite, laminates with cross direction 0°/90° and different thickness were also prepared. Steel bar frames with different thickness were used as a thickness gauge for precisely controlling the thickness of the composites to be a certain value. Specimens for property testing were cut from the composites along cross-laminated directions using a mold cutter and each sample had a splicing line at the center.

### 2.3. Mechanical Testing 

Tension and bending tests were conducted according to ASTM D638 and ASTM D790, respectively. For the tension test, the nominal specimen size was 165 × 13 mm^2^ and the cross-head speed was 5 mm/min. For the flexural tests, specimen size was 80 × 12.7 mm^2^ and a cross head speed of 5 mm/min was used. In total, 3 replicates (three specimens each) were tested. 

### 2.4. Water Uptake Testing

A series of 24 h water absorption and thickness swelling tests were conducted in accordance with ASTM D 570 specification at about 76.2 × 25.4 mm^2^ to evaluate the water sorption behavior. Three test bars were conducted for every composite sample at different junction conditions.

### 2.5. Scanning Electron Microscopy

The fracture surface of different splicing position and thickness of the composite specimens as well as the structure of the continuous laminates were observed via a scanning electron microscope (EVO-18 SEM, Zeiss, Jena, Germany) after sputter deposition of a thin conductive coating onto the samples (5 mm (L) × 5 mm (W) × Thickness).

## 3. Results and Discussion

### 3.1. Mechanical Properties

**Figure 4:**
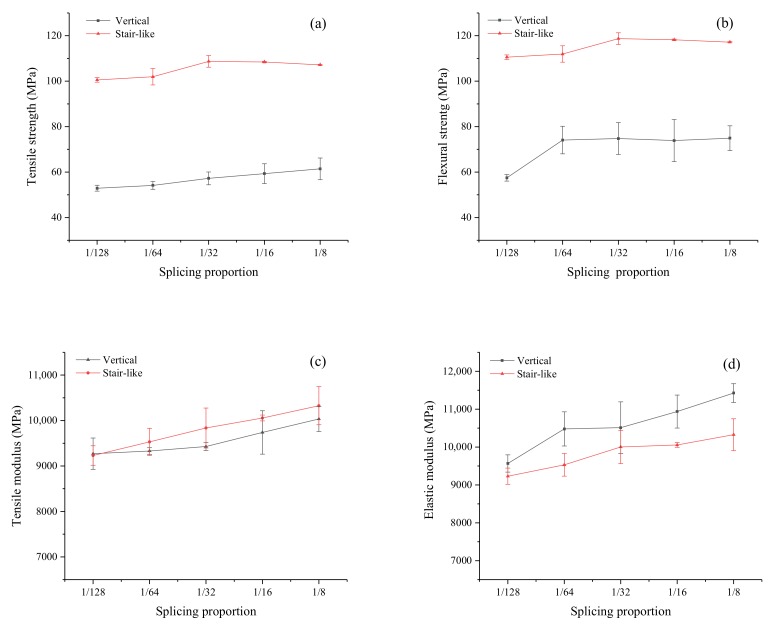
Effects of splicing proportion on tensile strength (**a**), flexural strength (**b**), tensile modulus (**c**) and elastic modulus (**d**) of composites with 0° direction.

**Figure 5:**
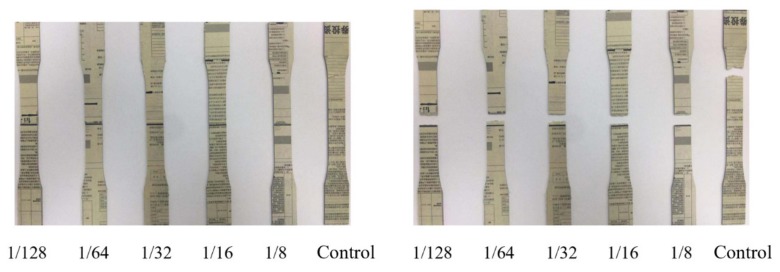
Specimens with different splicing proportions before (left) and after (right) tensile testing.

The effects of different splicing positions at the paper sheet length and width directions on the tensile and flexural strength of the composites are shown in Figure 4. It could be observed that both the tensile and flexural strength of the samples with a vertical splicing junction were lower than those of samples with a stair-like splicing junction. The junction uniformly distributed along thickness for the stair-like splicing pattern meanwhile the junction repeated at the same vertical position along thickness for the vertical splicing pattern. The junction between the laminar was a defect to break the continuity of reinforcement, which would cause stress concentration and deficient stress transfer. It was reported that the stress at the splicing positions were transferred by the matrix and the mechanical strength of the composites were increased with the increasing splicing distance [18]. The paper sheet acted as a reinforcement and the HDPE film was like a binder. The splicing distance between the two adjacent junctions determined the overlap area to affect the weakening effect on the mechanical properties of the composite. Generally, the mechanical strength of the composites will increase with increasing the splicing length, and there will be a critical length where the strength increase is no longer significant [19]. Both the tensile and flexural strength increased with the increase of the splicing distance. However, the increase became flat when the splicing distance reached the proportion of 1/32, where the overlap lengths at the length and width directions were 24 and 16 mm, respectively. The fractures were mainly located at the center of the specimens, where there was one of the splicing positions (Figure 5). To minimize the weakening effects of the junction on the mechanical properties of the composite, the overlap size should be greater than the critical proportion of 1/32 during the stacking procedure for a large-scaleale composite. When the splicing distance was the ratio of 1/32, the tensile and flexural strength of the composites were 109 ± 2.6 and 119 ± 2.6 MPa, respectively, which were very close to those of the control samples without splicing. The elastic moduli of stair-like splicing composites were lower than those of vertical splicing composites, which were thought to be due to the damage mode of different splicing patterns. For stair-like splicing composites, samples in flexural testing were progressively damaged with a large deformation. Brittle fracture occurred since there was a stress concentration at the vertical splicing position of the vertical splicing sample. Therefore, the elastic moduli of the composites with a stair-like splicing pattern were much lower.

To engineering design the physical and mechanical properties of the composite, the mechanical properties of the laminated composites with cross laminated structure at splicing distance of the 1/32 proportion were presented in Table 2. All composite samples with 0° direction exhibited extremely high mechanical performances. The tensile strength and modulus of NP sheet at 0° directions were measured to be 52 ± 2.4 and 6909 ± 426 MPa, respectively. However, these values were up to 73 ± 2.5 and 9166 ± 484 MPa after 25 min of hot pressing at 160 °C. It was thought that high mechanical performances were attributed to the compacted structure, and that the dense structure of the laminated composites was favorable to improve the mechanical strength because the enhanced friction among fibers increased the difficulty of fibrous pulling out. Besides, the perfect permeation by HDPE can also improve the stress transfer. The paper sheet, acting as a reinforcing material, had stronger strength at the length direction so that the mechanical strength of composites, which the paper sheets were stacked along their length directions, was two to three times higher than those of composites with the cross laminated structure. The strength and modulus of the control sample with the cross laminated structure had almost average values between the value of the length direction and the value of the width direction. It was reported that the modulus and strength of the composite were decreased as the fiber angle increased, which could be described by the Hankinson model [20]. However, the strength and modulus of the cross laminated composites with splicing had almost no change compared to those values of the composite at weak axis direction. The reason was thought to be that the weakening effects of the splicing junction were dominant to govern the mechanical properties of the composite. 

**Figure 6:**
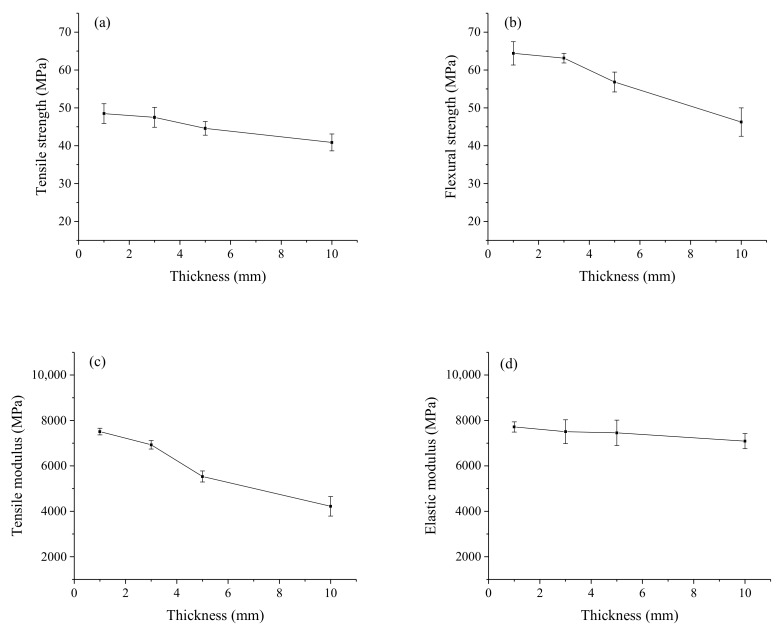
Effects of thickness on tensile strength (**a**), flexural strength (**b**), tensile modulus (**c**) and elastic modulus (**d**) of composites with vertical splicing.

The effects of the composite thickness on the tensile and flexural properties are shown in Figure 6. The thin sample had higher tensile strength and modulus values of 48 ± 2.6 and 7510 ± 147 MPa comparing to the thick sample having the tensile strength and modulus values of 41 ± 2.2 and 4220 ± 429 MPa. Generally, higher orientation can be obtained usually in the case of thinner samples, which results in a certain increase of the properties and its anisotropy. The results indicated a negative size effect on the mechanical properties of the composite. The negative size effect was due to the weakest link theory, according to the assumption that failure-inducing defects increased when the volume of the test specimen increased for the fiber reinforced composites [21]. Herein, pores and voids acted as the structure defects of the composites that were mainly distributed inside the NP sheet, bonding interface (as shown in Figure 9) and splicing cracks, which occurred unavoidably in hand stacking and splicing. The accumulation of these failure-inducing defects led to a high stress concentration with the increase of the thickness. As NP layers were compressed, part pores and voids were occupied by melted HDPE. The volume of residual pores and voids (*V_p_*) can be calculated by Equation (1):

(1) $$V_{p}=\frac{m_{c}}{\rho_{c}}-\frac{m_{NP}}{\rho_{NP}}-\frac{m_{HDPE}}{\rho_{HDPE}}$$

where *p* referred to the pores and voids and *c* was the composite. The density of compacted NP sheets ($\rho_{NP}$) was calculated to 1.75 g/cm^3^ according the amount of CaCO_3_ (*ρ* = 2.71 g/ cm^3^) and organic material (*ρ* = 1.50 g/ cm^3^ in literature). The volume fraction of residual pores and voids ($P_{c}\%$) values ranging from 6.5 to 8.4 vol% for composites samples with thickness increased from 1 to 10 mm, obtained from Equation (2):

(2) $$P_{c}\%=\frac{V_{p}}{V_{c}}\times 100\%$$

The superposition effect of the failure-inducing defect and the weakest link at the splicing position caused the flexural strength and the elastic modulus to significantly decrease from 64 ± 3.1 to 46 ± 3.8 MPa and from 7713 ± 224 to 7089 ± 334 MPa, respectively. The partial delamination may happen at the splicing position due to the existence of shear stresses during the flexural test. Additionally, the heating temperature inside the laminate was transferred by the surface layers. To ensure a sufficient compaction and infiltration of each layer, the time to press the thick composite was more than that to press the thin composite, which would cause the excessive permeation of HDPE into NP sheets at the laminar interface. It was found in the previous study that the excessive permeation of the HDPE would destroy the mechanical interlock between the NP fibers to break the continuity and integrity of the NP sheet, which would cause a negative impact on the enhancement of the NP sheets [22]. 

### 3.2. Water Adsorption and Thickness Swelling

**Figure 7:**
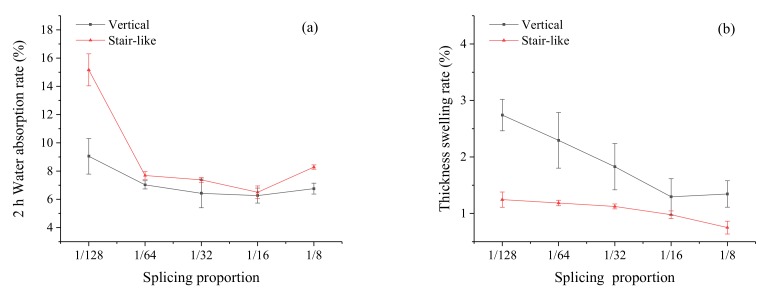
The 2 h water absorption (**a**) and thickness swelling rate (**b**) of composites with different splicing proportions.

**Figure 8:**
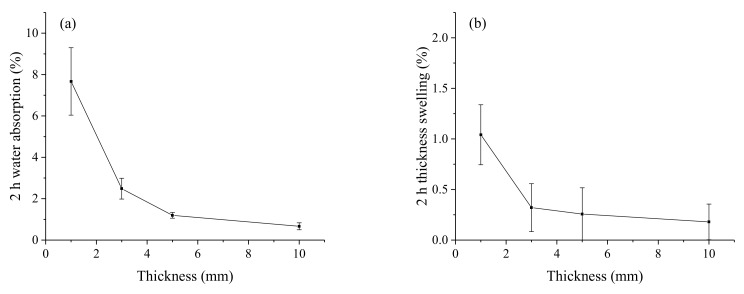
The 2 h water absorption (**a**) and thickness swelling rate (**b**) of composites with different thickness.

Water absorption and thickness swelling of the splicing laminates were tested in accordance with ASTM D570. It was shown in Figure 7 that samples showed relatively high values of water absorption and thickness swelling rates, which was attributed to the high hydrophobicity of cellulose, porosity of the NP sheets, and weak adhesion between the paper fiber and HDPE. The 2 h water absorption and thickness swelling of the splicing composite reduced significantly with the increase of the splicing proportion. The 2 h water absorption and thickness swelling of the vertical splicing composite were 6.42% ± 1.02 % and 1.82% ± 0.41 % meanwhile the values of the stair-like splicing composite were 7.38% ± 0.17 % and 1.12% ± 0.04 %, close to the values of the control laminates (5.87% ± 1.08 % and 1.49% ± 0.51 %) when the splicing proportion increased 1/32. It was also observed in the Figure 8 that the water absorption rate of the vertical splicing composites was lower than that of the stair-like splicing composite, meanwhile the thickness swelling rate of the vertical splicing composites was higher. It was thought that the uniform distribution of the splicing positions could improve the dimensional stability of the composite, although it could not reduce the water absorption. 

The effects of thickness on the water up-taking behavior are shown in Figure 8. Similarly, all samples showed unexpected high values of water absorption and thickness swelling rate. Since the NP layers stacked alternately with the HDPE films, it can be considered that the moisture was mainly transferred along the transverse direction rather than the thickness direction. The HDPE layers between the NP layers performed as a barrier to water transfer, and the cross sections of the samples were not protected from water uptake, leading to accelerated water absorption. The 2 h water absorption and thickness swelling of the composites decreased with the increase of the laminated thickness. The 2 h water absorption and thickness swelling of composite samples were 0.67% ± 0.17 % and 0.18% ± 0.18 % when the laminated thickness increased from 1 to 10 mm. It was thought that the excessive permeation of the HDPE would destroy the continuity of paper fibers to reduce the reinforcement of the paper sheet on the mechanical properties of the composites. However, the excessive permeation of the HDPE would form a hydrophobic coating to reduce water up-taking sites of the composites.

### 3.3. SEM Analysis

**Figure 9:**
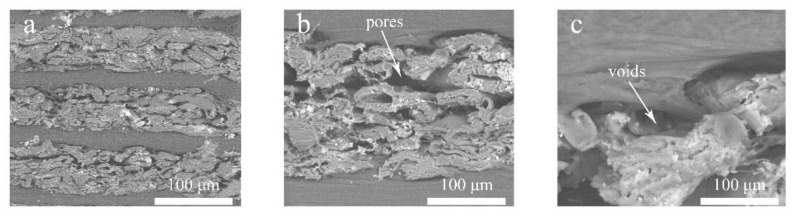
Microstructures of composites samples with 1/32 splicing proportion and 5 mm thickness: (**a**) cross section of composites; (**b**) pores in NP layer; (**c**) interface between NP layer and HDPE layer.

**Figure 10:**
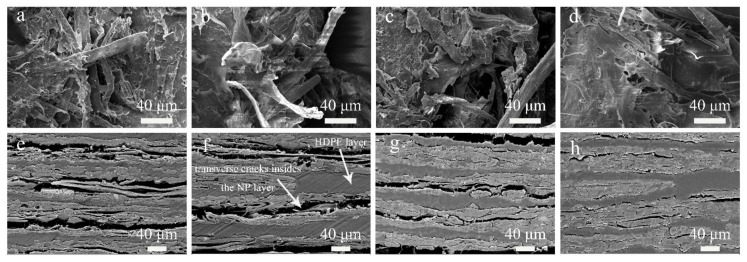
SEM of cross-section of composites with different splicing proportion in 0° direction (**a**) proportion of 1/128; (**b**) proportion of 1/64; (**c**) proportion of 1/32; (**d**) proportion of 1/16, and composites with different thickness: (**e**) 1 mm; (**f**) 3mm; (**g**) 5 mm; (**h**) 10 mm of vertical splicing composite.

The composite microstructures with 1/32 splicing proportion and 5 mm thickness are shown in Figure 9. The NP layer has lots of pores, voids and transverse cracks. The interface between NP and HDPE were clearly observed due to a weak bonding interface because no coupling agents and any surface treatment of the NP were used. The voids can also be seen between the NP and HDPE layers. It was found that the interfacial bond between NP sheet and HDPE layers was the mechanical interlocking rather than the chemical binding, where the HDPE acted as an adhesive so that fibers were bonded tightly to form a dense structure (Figure 10d). The mechanical interlocking performs desirable bonding capability where the transverse cracks and delamination mainly occur inside the NP layer rather than the bonding interface (Figure 10e–g). 

The fibers were completely pulled out at the fracture surface when the splicing proportion was less than 1/32 (Figure 10a–c) because of the absence of an effective stress transfer at the splicing position. A flat tensile fracture was observed in Figure 10d when the splicing proportion was 1/16, which indicated an increase of the splicing proportion could reduce the stress concentration. 

There were obvious transverse cracks and delamination insides the NP layers of thin composites, while a fully filled and highly compacted structure was obtained for composites with 10 mm thickness. As mentioned before, more hot-pressing time was required in thick composites to ensure a full filling inside the NP layers. It can be considered that there will be a critical thickness that the pores and voids inside the NP layers will be filled perfectly by melted HDPE and no delamination will occur in the composites. The continuity between the NP fibers was gradually destroyed by the HDPE. The above observed microstructure details could confirm that the excessive permeation of HDPE caused the reduction of mechanical properties and water absorption. 

## 4. Conclusions

The effects of the splicing pattern on the physical and mechanical properties of the full-scale recycled newspaper/HDPE laminated composite were studied. The main findings were summarized as follows:

1. The laminated composite with a stair-like splicing pattern showed a better physical and mechanical performance comparing to the laminated composite with a vertical splicing pattern.
2. The flexural strength and the elastic modulus of the laminated composite significantly decreased due to the superposition effect of the failure-inducing defect and the weakest link at the splicing position.
3. The distance between the adjacent two junctions should be greater than the critical proportion, which was 1/32 of the length at longitudinal direction, to ensure efficient stress transfer.

## Figures and Tables

**Table 1:** Different distances between the adjacent two junctions.

Proportion	Actual Distance	
	Width (0°) /mm	Length (90°) /mm
1/128	4	6
1/64	8	12
1/32	16	24
1/16	32	48
1/8	64	96

**Table 2:** Mechanical properties of laminated composites with different stacking directions.

Composite Structure	Stacking Direction	Tensile Strength (MPa)	Tensile Modulus (MPa)	Flexural Strength (MPa)	Elastic Modulus (MPa)
Vertical Splicing	0°	67 ± 2.2	9580 ± 28	88 ± 3.3	10511 ± 445
	90°	36 ± 1.0	4450 ± 89	56 ± 3.8	4283 ± 340
	0°/90°	39 ± 2.3	7461 ± 306	88 ± 1.9	7821 ± 640
Stair-like Splicing	0°	109 ± 4.2	9836 ± 411	119 ± 7.1	10002 ±346
	90°	40 ± 1.8	4393 ± 162	61 ± 0.9	4481 ± 378
	0°/90°	42 ± 1.9	5231 ± 256	67 ± 2.6	5921 ± 537
Continued Laminates	0°	107 ± 0.6	9902 ± 94	123 ± 1.3	10697 ± 887
	90°	41 ± 0.6	4551 ± 76	66 ± 1.4	5006 ± 110
	0°/90°	74 ± 0.7	7355 ± 104	100 ± 3.2	7540 ± 712
